# Internal pH regulation facilitates *in situ* long-term acclimation of massive corals to end-of-century carbon dioxide conditions

**DOI:** 10.1038/srep30688

**Published:** 2016-08-01

**Authors:** M. Wall, J. Fietzke, G. M. Schmidt, A Fink, L. C. Hofmann, D. de Beer, K. E. Fabricius

**Affiliations:** 1 GEOMAR Helmholtz Centre for Ocean Research Kiel, Germany; 2 Alfred-Wegener Institute, Helmholtz Centre for Polar and Marine Research, Bremerhaven, Germany; 3 Max-Planck Institute for Marine Microbiology, Bremen, Germany; 4 Marine Botany, Bremen Center for Marine Research and Education, University of Bremen, Germany; 5 Australian Institute for Marine Science, Townsville, Australia

## Abstract

The resilience of tropical corals to ocean acidification depends on their ability to regulate the pH within their calcifying fluid (pH_cf_). Recent work suggests pH_cf_ homeostasis under short-term exposure to pCO_2_ conditions predicted for 2100, but it is still unclear if pH_cf_ homeostasis can be maintained throughout a corals lifetime. At CO_2_ seeps in Papua New Guinea, massive *Porites* corals have grown along a natural seawater pH gradient for decades. This natural gradient, ranging from pH 8.1–7.4, provides an ideal platform to determine corals’ pH_cf_ (using boron isotopes). *Porites* maintained a similar pH_cf_ (~8.24) at both a control (pH 8.1) and seep-influenced site (pH 7.9). Internal pH_cf_ was slightly reduced (8.12) at seawater pH 7.6, and decreased to 7.94 at a site with a seawater pH of 7.4. A growth response model based on pH_cf_ mirrors the observed distribution patterns of this species in the field. We suggest *Porites* has the capacity to acclimate after long-time exposure to end-of-century reduced seawater pH conditions and that strong control over pH_cf_ represents a key mechanism to persist in future oceans. Only beyond end-of-century pCO_2_ conditions do they face their current physiological limit of pH homeostasis and pH_cf_ begins to decrease.

Tropical corals are the foundation species for coral reefs, the most diverse marine ecosystems in the world. The persistence of a species-rich reef-associated community will depend on the ability of corals to maintain net growth under future *p*CO_2_ conditions. To date our understanding of the fate of corals in the face of ocean acidification is based on controlled laboratory studies^1^,^2^, mesocosm studies mimicking coral community composition^3^,^4^,^5^, and field sites that function as natural ocean acidification analogues^6^,^7^,^8^. These efforts have provided strong evidence that many tropical corals will respond to future predicted *p*CO_2_ conditions with a decline in growth. However, corals actively establish a proton (H^+^) gradient by pumping protons out of the calicoblastic space between tissue and skeleton where calcification takes place, maintaining the pH of the calcifying fluid (pH_cf_) well above seawater pH (pH_T_)^9^,^10^. Therefore, the aragonite saturation state at the site of calcification is elevated relative to seawater, which likely fosters calcification.

The magnitude of the pH_cf_ up-regulation can be derived indirectly by measuring the skeletal boron isotopic composition (δ^11^B) – an established pH proxy that appears to vary with pH_cf_ at the calcification site^9^,^11^,^12^,^13^,^14^. Thus, it can be and is already used to determine the corals’ ability to elevate the pH_cf_ at the site of calcification^11^,^14^,^15^,^16^,^17^. Culturing experiments have revealed that a reduction in seawater pH_T_ is not directly reflected in the skeletal boron isotopic composition^11^,^12^,^13^, as the decline in skeletal δ^11^B, and hence internal pH_cf_, is less than the change in seawater pH_T_. At low seawater pH_T_, internal pH_cf_ is still elevated compared to seawater pH_T_ (up-regulation intensity, where ΔpH = pH_cf_ − pH_T_^18^,^19^,^20^), but it does not reach those internal pH_cf_ levels observed under control conditions^11^,^18^. Based on the observed relationship between internal pH_cf_ and seawater pH_T_ from laboratory studies, McCulloch *et al*.^14^ projected a continuous decline in growth under ocean acidification using their internal pH regulation and abiotic calcification (IpHRAC) model. The projected decline is species-specific, with massive *Porites* being regarded as a rather robust coral taxon. A recent short-term study, however, observed that corals exposed to reduced seawater pH_T_ conditions in an *in situ* mesocosm experiment can maintain their internal pH_cf_ irrespective of seawater pH_T_ down to pH_T_ 7.74^16^. While these data provide hope for coral persistence in the future, they cannot tell whether corals can maintain their internal pH_cf_ in the long-term in their natural environment or if they are able to acclimate after long-term exposure to future ocean seawater pH_T_.

Volcanic carbon dioxide seeps in Milne Bay Province, Papua New Guinea (PNG) represent an ideal natural laboratory to investigate the effect of a seawater pH_T_ gradient on coral skeletal δ^11^B and pH_cf_ upregulation. A previous study found that at these seeps, massive *Porites* corals dominate coral reefs at seawater pH_T_ levels projected for the end of the century (~7.8), and growth rates are similar compared to adjacent control sites^6^. At a seawater pH_T_ of 7.7, reef formation ceases, and only a few scattered colonies of massive *Porites* are found close to a major seep site where seawater pH_T_ is severely reduced (Supplementary Fig. S1). No corals are found below a seawater pH_T_ of ~7.4, where seagrasses dominate the environment^6^. These distributional data contrast with projections based on the previously mentioned laboratory findings^1^, but allow testing of whether or not pH_cf_ up-regulation is a key mechanism that allows *Porites* to dominate the PNG seeps and maintain pH_cf_ homeostasis during their lifetime.

To investigate the corals’ ability to regulate their internal pH_cf_ *in situ* along a seawater pH_T_ gradient, we studied skeletal samples of massive *Porites* colonies from the CO_2_ seeps in PNG. Fourteen corals were sampled from four sites, namely a control site (8.1 pH_T_), intermediate site (7.9 pH_T_), low pH_T_ site (7.6 pH_T_), and extreme site (7.4 pH_T_; Supplementary Fig. S1, Table S1,3). We tested whether the IpHRAC model^14^ can be used to reproduce the observed pattern in *Porites* distribution and growth by inferring the internal pH_cf_ from the skeletal boron isotopic signature. We used high-resolution boron measurements to address whether strong natural variations in seawater pH_T_ (as observed at the seep sites) are reflected in the skeletal boron isotopic signature. This has important implication for the use of δ^11^B to distinguish between sites of high seawater pH_T_ variability (e.g. internal wave influenced reefs, upwelling) and sites with more stable conditions^21^,^22^.

## Results and Discussion

**Figure 1:**
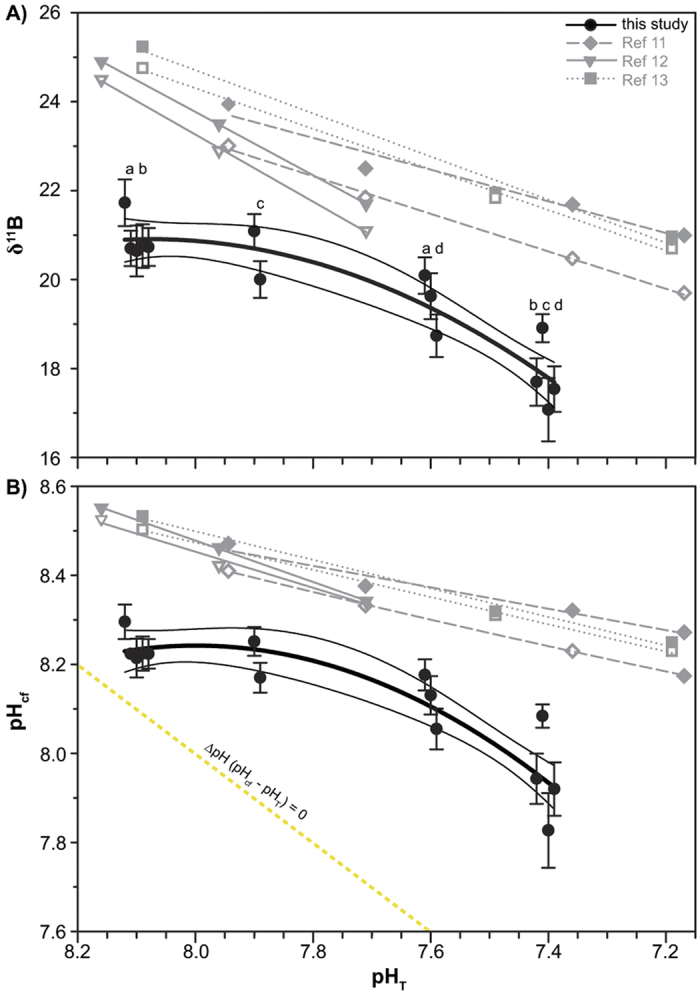
Boron isotopic signature and corresponding internal pH_cf_ of *Porites* corals from the Papua New Guinea (PNG) *p*CO_2_ seeps. (**A**) Average δ^11^B values measured in 14 massive *Porites* coral skeletons collected along a seawater pH_T_ (pH in total scale) gradient at the PNG seeps. (**B**) Coral skeletal δ^11^B signature translated into internal calcifying fluid pH (pH_cf_). Black filled circles and error bars are means ±1 SE per colony (n = 15–20 samples per colony). Individual colonies at each site are offset horizontally for clarity. Black lines: Regression analysis following a second-order polynomial fit (thick black line) with 95% confidence interval (thin black lines). Grey symbols and lines represent literature data of laboratory findings for tropical corals^11^,^12^,^13^ (^11^open symbol: *Stylophora pistillata* lateral growth and filled symbol: *Stylophora pistillata* apical growth, ^12^open symbol: *Acropora nobilis* and filled symbol: *Porites cylindrica*, ^13^open symbol: *Stylophora pistillata* and filled symbol: massive *Porites* sp.). Letters (a, b, c and d) indicate results of the post hoc test when there was a significant site effect (p < 0.01). Statistical test can be found in the supplements Table S4. Yellow dashed lines indicate internal pH conditions when organisms are not up-regulating pH_cf_.

**Figure 2:**
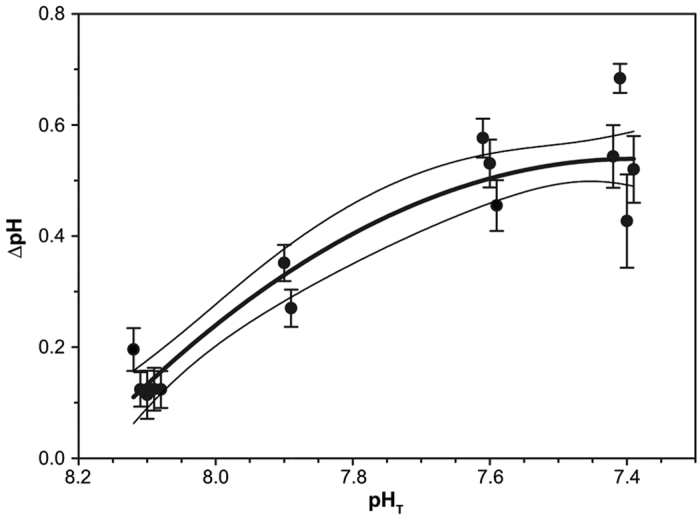
Massive *Porites* corals pH up-regulation. Internal pH up-regulation intensity of corals collected (ΔpH) along a natural seawater pH (pH_T_ in total scale) gradient. Symbols display mean ± SE values for each coral colony collected at four sites with known pH_T_ conditions. Solid black lines indicate regression analysis following a second-order polynomial fit (thick black line) with 95% confidence interval (thin black lines).

We derived the first δ^11^B-pH_T_ relationship for tropical corals collected along a natural pH_T_ gradient (Fig. 1A). The observed relationship of δ^11^B against the mean seawater pH_T_ values recorded at the four sites (Supplementary Figs S3 and 4, Table S3) differs from previous laboratory findings (Fig. 1A). The δ^11^B values of the five corals from the control site agreed well, but could not be distinguished from those corals collected at the intermediate site, while those from the low pH_T_ site were lower than and significantly different from the control site (mean of all colonies per site ± s.e.m.: control site 20.91‰ ± 0.26 and low pH site 19.48‰ ± 0.40, respectively, Supplementary Table S5). At the extreme site the δ^11^B values were significantly lower than δ^11^B at all other sites (Fig. 1A, Supplementary Tables S4,5). In contrast to laboratory studies (using the same genus *Porites* and two other genuses namely *Acropora* and *Stylophora*, Fig. 1A), the here observed trend does not allow the reconstruction of seawater pH. This is similarly to a recent study^16^ (see also Supplementary Material: *“Average boron isotopic signature, variability and corresponding internal calcifying conditions”*). Also our study and this recent work^16^ show that variations between individuals are often greater than the effect of external environmental conditions on the boron isotopic composition (e.g. individual differences at control = 1‰ and intermediate site = 1.09‰, compared to an average difference of 0.34‰). Corresponding pH_cf_ values suggest that the corals’ internal pH_cf_ remained within a narrow range, with mean values ranging from 8.30 to 7.83, while the seawater pH_T_ changed from 8.1 to 7.4 (Fig. 1B), as confirmed by direct pH_cf_ measurements^9^. This underlines a strong physiological control on their internal pH_cf_ irrespective of seawater pH_T_. The pH up-regulation (∆pH) effort was significantly higher at all seep-influenced sites compared to the control site (mean of all colonies per site ± s.e.m.: control site 0.14 ± 0.02 pH units, intermediate site 0.31 ± 0.04, low pH_T_ site 0.52 ± 0.04 and extreme site 0.54 ± 0.05, respectively; Supplementary Table S4,5). The highest mean **Δ**pH up-regulation observed in any of the studied colonies was 0.68 ± 0.03 (Fig. 2).

The corals in this study were growing within a few hundred meters distance from each other, under similar physicochemical settings excluding *p*CO_2_ (e.g. similar water flow, salinity, temperature, nutrient levels, total alkalinity)^6^,^23^. Under long-term exposure to these natural environmental conditions, the corals showed the ability to compensate for reduced external seawater pH_T_ by increasing internal pH_cf_ up-regulation. Combined with results from a recent study^16^, our results suggest corals can maintain pH_cf_ homeostasis and highlight that even corals exposed to *p*CO_2_ conditions predicted for the end of the century for their entire lifetime can maintain internal pH_cf_. Only beyond this threshold do they face their current physiological limit, where pH_cf_ begins to decrease.

We calculated relative rates of calcification based on our internal pH_cf_ values using the IpHRAC model^14^: G = k*(Ω_cf_ − 1)^n^ (see McCulloch *et al*.^14^, and Materials and Methods for more details). Fabricius *et al*.^6^ only measured growth at vent sites with seawater pH_T_ levels not lower than 7.75 (expected seawater pH_T_ values for the end of the century), not covering the seawater pH_T_ range of this study. We used their measured growth ratio and compared it to the relative growth rate calculated by the IpHRAC model based on our derived pH_cf_. The similarity in growth (G) between our intermediate site and the control site (G_intermediate_/G_control_ = 1.23 to 0.91; Supplementary Table S6) corroborates the lack of calcification response observed in a previous study^6^. Hence, we used the model to extrapolate growth for the other two sites (low pH_T_ and extreme site). Calcification rates at the low pH_T_ site are still similar to present day rates and become reduced at the extreme site. Overall, our modelled growth response mirrors the coral distribution observed at the PNG sites.

**Figure 3:**
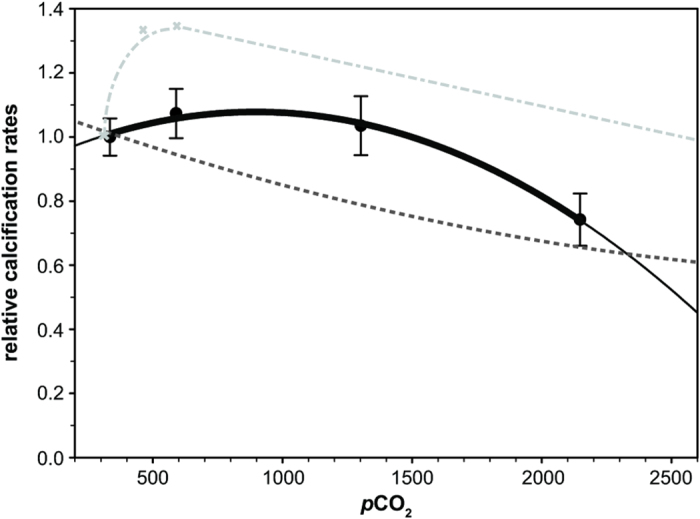
Growth response modelled for massive *Porites* corals at the Papua New Guinea seeps. The modelled growth response displays relative changes in calcification rate (relative calcification rate = mean control/mean site). Black circles and error bars represent means ±1 SE per site and the black solid line indicates a second-order polynomial fit for the growth model in this study. The growth response curve is compared to published growth responses: McCulloch *et al*.^19^ (dark grey dashed line) and Castillo *et al*.^24^ (light grey dashed line).

The IpHRAC model from McCulloch *et al*.^14^ for *Porites* (Fig. 3) suggests a continuous decline in growth and contrasts to the model derived using our data from the PNG seeps. Here we found a similar growth rate for control, intermediate and low pH_T_ site before growth rates decrease to the extreme site (Fig. 3). The **Δ**pH up-regulation intensity potentially reaches a physiological limit and becomes energetically expensive at the extreme site. This site corresponds to the limit of *Porites* occurrence at the PNG seeps, beyond which the coral do not grow. Corals at seawater pH_T_ levels of 7.9 potentially acclimate to these pCO_2_ conditions by enhancing internal pH_cf_ up-regulation. Laboratory studies have also shown a curvilinear growth response^9^,^24^, even with similar growth rates at a *p*CO_2_ of 2553 ppm (pH_T_ = 7.32) compared to pre-industrial levels (Fig. 3^24^). In the latter study^24^, they did not test whether internal pH_cf_ was similarly elevated at *p*CO_2_ 324 and 2553 ppm. Our study and a recent pH_cf_ homeostasis hypothesis^16^ would indicate that pH_cf_ in corals exposed to both treatments should be similarly elevated, but this still needs to be validated for the experiment by Castillo *et al*.^24^. Considering the short duration (95 days) of their experiment, it is questionable whether the corals would be able to maintain calcification at 2553 ppm CO_2_ for longer periods of time, considering the expected increase in energy demand at ecological time scales. Our calcification model (Fig. 3) suggests that even the very robust *Porites* corals would have reduced rates of calcification when exposed to levels that are far beyond those projected for the end of the century for a lifetime. Calcification is an energy expensive process and hence, the increased up-regulation at the seep sites requires more energy that must be provided in order for the corals to acclimate. The expected increase in seawater dissolved inorganic carbon in future oceans may enhance photosynthesis, and consequently provide more energy to the corals at the intermediate and low pH_T_ sites to cover their increased daily budget without negative consequence^24^,^25^. However, the increase in photosynthesis might not be sufficient to maintain a high pH_cf_. Furthermore, it is not known what other physiological and metabolic trade-offs the corals may face, potentially affecting the calcification response and also their viability. Here we support previous findings that internal pH_cf_ up-regulation mitigates ocean acidification^9^. Thus, pH up-regulation represents a mechanism that can make corals more resilient to future pCO_2_ conditions^14^. Venn *et al*.^9^ cultured corals under various pCO_2_ levels and observed a similar calcification response as modelled here. However, in contrast to our observations their directly measured internal pH_cf_ decreased with external seawater pH changes. They explored two potential models (extended models of McCulloch *et al*.^14^) and tested whether they can explain their observed calcification rates based on their internal pH_cf_ values. One model assumes constant energy investment and proton removal rate, and the second model is based on a variable proton removal rates. Their second model more closely represents their corals’ response with an initial increase and then a decrease in proton removal rate. We do not have independent measurements of calcification rates for the low pH_T_ and extreme sites. Such data would allow to test whether the modelled growth values (based on mainly the boron derived internal pH_cf_) also match measured growth data for these sites as they did for the intermediate site. What we wanted to point out here is that while for our intermediate site boron derived internal pH_cf_ and modelled growth agree, internal pH_cf_ is likely not the only determining factor for calcification rates. Hence, the variable proton removal model revealed a very important aspect: to fully understand the calcification response we need to constrain more than just internal pH_cf_ and calcification rates. Studies investigating gene regulation variation as a consequence of increasing pCO_2_ conditions^26^,^27^ indicated that full suite of processes are potentially affected by ocean acidification and can affect calcification rates. For instance, after short-term exposure to near-future seawater pH_T_ conditions, corals responded with an up-regulation of genes involved in ion transport (in particular Ca^2+^-transporters like Ca-ATPase that also affects internal pH_cf_, but also bicarbonate transporters)^26^. Such a response might help to maintain the internal pH_cf_ and calcification rate. In the same study^26^, short-term exposure of corals to pH_T_ 7.2 resulted in a down-regulation of ion transporters and potentially can explain physiological limits in growth. Ocean acidification also can affect a wide range of cellular processes that are not directly linked to biomineralization^27^. These studies indicate the need for a more comprehensive approach combining physiological and transcriptomic investigations with ecological and geochemical studies.

Natural analogues to ocean acidification, such as the PNG seeps, provide unique opportunities for studying the potential effects of elevated *p*CO_2_ on coral reefs, but they also have limitations, e.g. strong fluctuations in pH_T_/*p*CO_2_ and close connectivity to undisturbed areas that supply propagules. The physiological consequences of such strong pH_T_ fluctuations are still not fully understood. Recent studies have shown that growth in coral recruits was higher under fluctuating *p*CO_2_ conditions than under constant reduced pH_T_^28^, and that exposure to strong temperature variations resulted in an improved stress-resistance^29^,^30^ and faster acclimation^31^ in corals. Similarly, the hypothesized pH homeostasis observed during the free ocean carbon enrichment (FOCE) experiment, argues that the seasonal seawater pH_T_ variations the corals are facing are a driver for stronger control on their internal pH_cf_ environment^16^. Thus, the fluctuating conditions could potentially foster acclimation to low seawater pH_T_. Daily swings in *p*CO_2_ or strong fluctuating seawater pH_T_ conditions are not unusual in coral reefs^21^,^22^,^32^,^33^. At the CO_2_ seeps in PNG, *Porites* is able to cope with the projected near-future increase in *p*CO_2_, in contrast to most other coral species, including the structurally complex species that form the habitat for many reef-associated organisms^34^. Responses to *p*CO_2_ also vary between regions with naturally reduced seawater pH_T_^7^,^35^,^36^, as changes in seawater pH_T_ are not the only factor in the field and act in concert with the full suite of environmental variability (e.g. seasonality, differences in current regimes, etc.). In addition, boron isotopic composition is highly variable at high spatial resolution^15^,^17^, and in our study, irrespective of seawater pH_T_ variability. This agrees with the conclusion that such δ^11^B variations reflect the effect of biological processes on skeletal isotopic composition rather than external seawater pH_T_ variations^15^. In particular, since the control site corals (where the seawater pH is stable) showed the same skeletal variations. Several factors are thought to contribute to these internal variations in pH_cf_, but they are not yet fully understood (see also Supplementary Material: *Average boron isotopic signature, variability and corresponding internal calcifying conditions*).

Our study shows that massive *Porites* will be able to persist in the oceans of 2100, due to observed similar growth rates to present day conditions^6^,^25^, enhanced photosynthesis^25^ and also its ability to maintain a high internal pH_cf_. All of these factors contribute to *Porites’* dominance at the Papua New Guinea CO_2_ seeps. Enhanced pH_cf_ up-regulation enables them to sustain their present day calcification rate up to *p*CO_2_ levels projected for the end of the century. From massive *Porites* at the PNG seeps, we have observed that this species has the potential to adjust and maintain their internal pH_cf_ even after lifetime exposure to increased *p*CO_2_. For other more sensitive corals, it needs to be elucidated whether or not they are able to maintain their internal pH_cf_. Our study underlines that conclusions projected from laboratory studies alone need to be treated with caution, and should be complemented by results from field studies. Together with a recent study we emphasize that seawater pH reconstruction from *Porites* need to be taken with caution. Both studies underline this genus ability to exert strong physiological control. Such local acclimations represent one possibility for resisting future changes. It is thus essential to understand what allows corals in a certain environment to acclimate, and whether other species in other regions have the same capacity to adjust to future changes.

## Material and Methods

### Site description and coral core collection

Fourteen coral cores were collected during three research cruises from four sites that differed in their seeping intensity: an extreme site, a low pH_T_ site, an intermediate site, and a control site (Supplementary Material: *sites* and *coral sample overview*, Table S1, Fig. S1). The seawater pH_T_ adjacent to the coral colonies was recorded with data loggers and total alkalinity (TA) measured in discrete water samples. The carbonate chemistry was calculated from seawater pH_T_ and TA for the four sites (Supplementary Material: *Seawater pH*_*T*_ *characterization at the collection sites* and *seawater carbonate chemistry*, Figs S3-4, Table S3).

### Sample preparation and analyses

Coral skeletons were bleached for 24 h, thoroughly washed with milli-Q and dried overnight at 50 °C. Subsequently, they were embedded in resin, cut along the growth axis, ground and polished. From long cores, a piece approx. 5 mm wide and 1 cm long oriented along the growth axis was prepared for boron analysis and carefully ground and polished. The δ^11^B composition was measured with a laser ablation multi collector inductive coupled plasma mass spectrometer (Thermo Fisher MC-ICP-MS AXIOM, connected to a UP193fx laser ablation system of New Wave Research, equipped with an excimer 193 nm laser) following the method by Fietzke *et al*.^37^ (Supplementary Material: *Boron isotopic signature*, Table S2).

### Data analyses

The data reduction followed Fietzke *et al*.^37^. This yielded one δ^11^B value per sample and session with an average precision of 1‰ (1 SD) for approx. 2.5 μg of carbonate sample. A minimum of 15 individual values of δ^11^B spread over the core surface from the upper few mm of each coral colony were measured to obtain a representative data set per sample. The data set reflects the high variability in δ^11^B for a single colony. For each individual δ^11^B value the internal pH_cf_ and ∆pH was calculated. Individual values per colony were averaged to yield values that reflect the average δ^11^B value, the average internal pH_cf_ and ∆pH (see below).

Each individual δ^11^B value was translated into internal pH_cf_ following equation (1) with a seawater δ^11^B_sw_ of 39.61‰^38^, a fractionation factor (α_B_) of 1.0272^39^ and pK^*^_B_ of 8.56^40^.


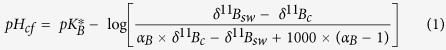


Following the method in Trotter *et al*.^18^, the superimposed physiological pH control was calculated using the equation:


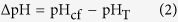


and related to the seawater pH_T_ to quantify the extent of the physiological control on the internal pH_cf_.

Calcification rate (G) was calculated following the McCulloch *et al*.^14^ IpHRAC model: G = k*(Ω_cf_ − 1)^n^. Seawater dissolved inorganic carbon concentration [DIC]_sw_ was calculated by the R package seacarb^41^ using the external seawater pH_T_ and the average total alkalinity (2272 μmol kg^−1^), salinity (34.5 ppm) and temperature (28.5 °C) measured at the sites^2^ (and Supplementary Table S3). Carbonate saturation state at the site of calcification (Ω_cf_) was calculated using seacarb by setting [DIC]_cf_ equivalent to 2*[DIC]_sw_^114^ assuming an elevated [Ca]^2+^ concentration of 11 mmol kg^−1^ and the average salinity and temperature. The modelled calcification response was calculated for constant temperature and with the temperature-dependent rate law constant k = 42.42 and reaction order constant n = 1.89 (applying the equations given in McCulloch *et al*.^14^: k = −0.0177*T^2^ + 1.47*T + 14.9 and n = 0.0628*T + 0.0985).

Data analysis and visualisation was done with R Studio version 3.0.1 (R Development Core Team, 2015). The regression analysis and growth model fit were done using a generalized linear model. An AIC criterion was used to find the best-fit comparing linear vs polynomial (2^nd^ and 3^rd^ order) fits. The software package visreg (2.0–4) was used to visualize the best fit.

## Additional Information

**How to cite this article**: Wall, M. *et al*. Internal pH regulation facilitates *in situ* long-term acclimation of massive corals to end-of-century carbon dioxide conditions. *Sci. Rep*. **6**, 30688; doi: 10.1038/srep30688 (2016).

## Supplementary Material

Supplementary Information
