# Supplementary material for: Internal pH regulation facilitates in situ long-term acclimation of massive corals to end-of-century carbon dioxide conditions
Source: Sci Rep. 2016 Aug 1;6:30688. doi: 10.1038/srep30688 (PMC4967918; doi:10.1038/srep30688)
Supplement: Supplementary Information [file srep30688-s1.pdf]

Supplementary material to

Internal pH regulation facilitates *in situ* long-term acclimation of massive corals to end-of-century carbon dioxide conditions

Wall M[1]*, Fietzke J[1], Schmidt GM[2], Fink A[3], Hofmann LC[4], de Beer D[3] and Fabricius KE[5]

[1]GEOMAR Helmholtz Centre for Ocean Research Kiel, Germany
[2]Alfred-Wegener Institute, Helmholtz Centre for Polar and Marine Research, Bremerhaven, Germany
[3]Max-Planck Institute for Marine Microbiology, Bremen, Germany
[4]Marine Botany, Bremen Center for Marine Research and Education, University of Bremen, Germany
[5]Australian Institute for Marine Science, Townsville, Australia

Materials and Methods:

- Sites:

The sites were chosen to span the entire seawater pH_T gradient at the Upa Upasina reef where corals are still present (published by Fabricius et al.[1] and reproduced here in Fig. S1a). We sampled at the spots marked in Fabricius *et al.*[1] in their Fig. 1d (here reproduced in Fig. S1a as C, I, E) and in addition, we sampled also a low pH_T site (Fig. S1a marked in the seawater pH_T map as L)).

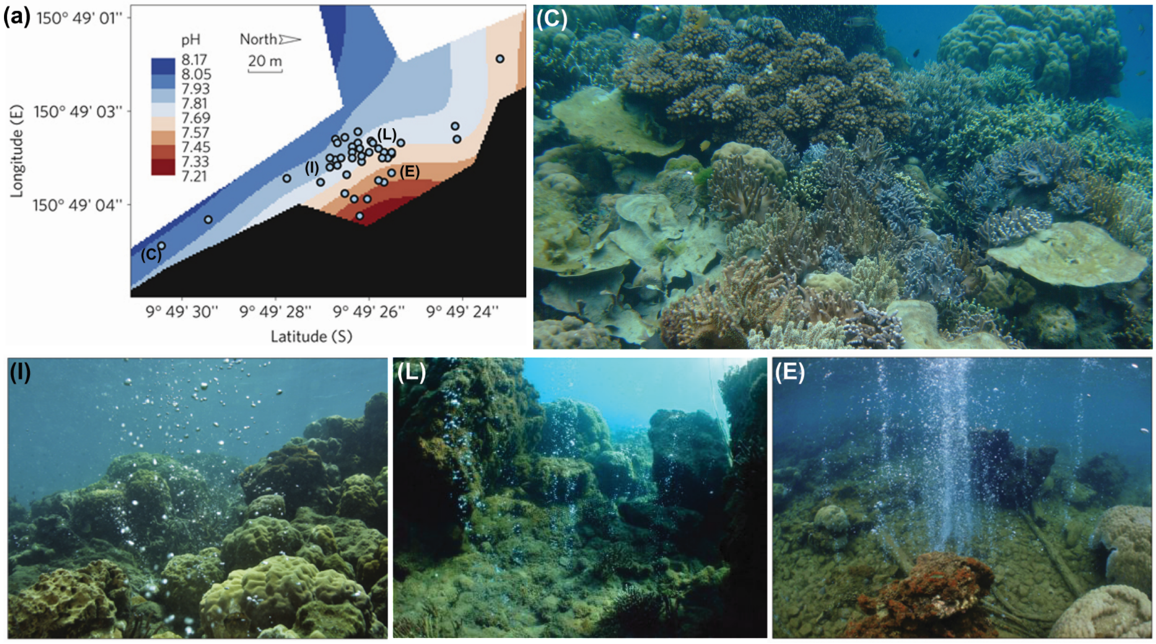

Fig. S1: a) The map displays the seawater pH gradient at the Upa Upasina Reef (modified from Fabricius *et al.*[1]). The sample sites are marked in the map and illustrated with the respective images: (C) control, (I) intermediate site, (L) low pH site, and (E) extreme site (Images: (C, I, E) from Fabricius *et al.*[1], (L) from K. Fabricius).

- ***Seawater pH_T characterization at the collection sites:***

The seawater pH (pH_T in total scale) was monitored at multiple locations within the seep site and at the control site by different instruments: a CTD (Seabird, SBE 19v2) equipped with a pH sensor (SBE 18), multi-channel loggers (XR-420, RBR Ltd.) with a pH sensor (AMT Analysenmesstechnik GmbH) and a SeaFET Ocean pH sensor (Satlantic). Prior to the deployment at the control site, the CTD was calibrated with NBS buffers (4.0, 7.0, 9.21; for slope evaluation of the calibration curve) and a TRIS buffer to derive seawater pH at total scale (pH_T)[2]. All instruments were placed at the control site side by side for cross-calibration. The CTD calibrated to total scale was used as reference, and all instruments were cross-referenced (deployed at the beginning at the control site) and corrected for their offset. The seawater pH_T measurements were taken next to the cores collected and during the respective field campaign of core collection. The extreme site was only measured during the last trip, when 3 of the in total 4 cores were collected. Discrete water samples were repeatedly collected and analyzed for total alkalinity. Total alkalinity was determined by gran titration following Dickson et al.[2].

- ***coral sample overview:***

The sampled coral cores (1 to 5 cm diameter), their location and the date of sampling are listed in Table S1. The seawater pH_T was monitored at the four sites (Fig. S1) during the three cruises. In Fig. S2 a overview of the different sample types are provided as well as the location of the measurements.

Table S1. Cores of massive *Porites* collected, with collection date and location ID.

Core ID	Year/month	Location ID
**C1A**	2010/08	C
**C2A**	2010/08	C
**C3A**	2010/08	C
**C13**	2013/06	C
**C14**	2014/11	C
**I1**	2013/06	I
**I2**	2013/06	I
**L1**	2013/06	L
**L2**	2014/11	L
**L3**	2014/11	L
**E1**	2013/06	E
**E2**	2014/11	E
**E3**	2014/11	E
**E4**	2014/11	E

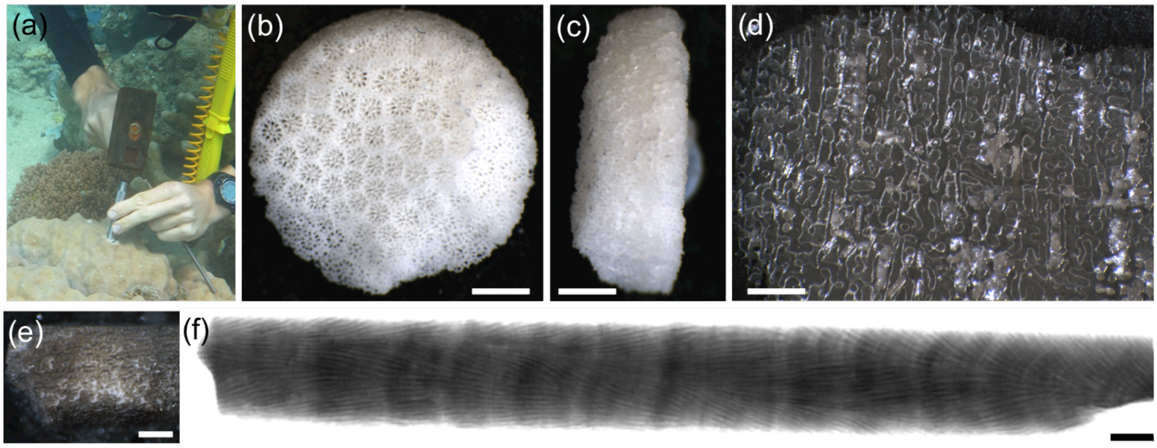

Fig. S2: The small coral cores were collected as shown in (a). The diameter was approx.. 10 mm ((b), scale bar 10 mm) and a height of 4-10 mm (example in (c) scale bar 2 mm). The individual measurements were spread over the upper part (few mm) of the core ((d), scale bar 2 mm). The long core (e,f) had a diameter of 5 cm and the outer part was sent to . In the upper part was prepared for LA-ICP-MS (e) after x-ray of the part of the coral core (f).

- *Boron isotopic signature:*

The Thermo Fisher AXIOM multi collector inductive coupled plasma mass spectrometer (MC-ICP-MS) connected to a laser ablation system of New Wave Research UP193fx was used to measure boron isotopes following the method of Fietzke et al.[3]. Faraday cups were used to collect data simultaneously for ^{10}B (amu10) and ^{11}B (amu11) in the outer most cups (L4 and H4). The resulting instrumental settings are summarized in Table S2 and were valid throughout all measurements performed. The cones were cleaned on a regular basis (every 2-4 days). The tubes going from the ablation cell to the plasma torch were checked for material deposition and cleaned by high flow rates overnight and/or mobilization of the debris by increased flow rates transporting it out of the tubes.
Prior to each measurement session, the standard and samples were pre-ablated to remove surface contaminations (spot size was used one size bigger than during analysis and 50shots per spot area = approx. 8-10 μm of surface material). A standard-sample-standard bracketing method was used. The data of one measurement session contained 5-6 brackets. Both ^{12}C and the variation of the standard for each session were used to check for instrument stability and contaminations. Sessions were excluded from further analysis when the standard drift was higher than the internal reproducibility of the standards (2SD of the session on the standards, $^{10}B/^{11}B$ of 0.00015 = 0.27‰). During each session approx. 2.5 μg of coral sample were ablated, corresponding to 0.13 ng of total B (using an average B concentration of 50 ppm for the coral carbonate).

Table S2: LA-ICP-MS settings.

**ICP-MS settings (AXIOM)**	
Cool gas (Ar)	14-16 l min-1
Auxiliary gas (Ar)	1.1-0.7 l min-1
sample gas (Ar)	1.05-0.75 l min-1
RF Power	1100-1250 W
Reflected	3-4.8 W
PowerAccelerating voltage	4972 V
Resolution	500res
Torch position	80-200
**Laser settings (UP193FX)**	

Ablation cell gas (He)	0.6-0.8 l min^{-1}
Spot size	35-50 μm
Fluence	1.8-2.6 J cm^{-2}
Repetition rate	30-40 Hz

The individual boron isotopic signature area sampled for an individual $\delta^{11}B$ data point was approx. 200-300 μm long with a spot diameter of 35-50μm. The spot were positioned at the edge of skeletal elements. Because the laser ablates in greater depth (~30μm) compared to SIMS (a few microns) we can not exclude that centres were not sampled in particular because in *Porites* centres are more discretely distributed and not always located in the central line[e.g. 4,5]. However, we expect that we sampled for all individuals a similar portion of centres of calcification. The length encompasses approx. 10 days up to two weeks of growth considering the average linear extension of the corals measured by Farbricius et al.[1] was ~ 1.2 cm and this corresponds to ~ 32 μm day^{-1}. Hence, they are within the temporal scale of the seawater pH_T monitoring.

The boron isotopic signature is reported in delta notation, normalizing the unknown sample relative to the known standard using the soda-lime glass NIST-SRM610 ($\delta^{11}B$=-0.55‰ ± 0.53) as bracketing standard during measurements:

$$\delta^{11}B = \left[\frac{(\frac{10B}{11B})sample}{(\frac{10B}{11B})Nist610}\right]-1$$

We compared individual spot measurements to investigate $\delta^{11}B$ variations between the control site and the different seep site locations that differ in their intensity of seawater pH_T variability[6] and also the relative changes between sites using average $\delta^{11}B$ values.

Results and Discussion:

- Seawater carbonate chemistry

The seawater pH_T is reduced where the almost pure volcanic CO_2 gas seeps through the seafloor. Persistent seeping has been confirmed for at least 70 years, but it has likely persisted for much longer[1]. At the control site, the seawater pH_T displayed only slight variations throughout the day, with an average seawater pH_T of 8.1 (SD = 0.10; Table S3). The pH_T fluctuations increase and the seawater pH_T become lower the closer to the major seeps. The intermediate site and low pH_T site showed average seawater pH_T values of 7.9 (SD = 0.20) and 7.6 (SD = 0.17), respectively. Seawater pH_T at the seep sites revealed strong variations in seawater pH_T at time scales of hours (Fig. S3-S4). During increased tidal or wind driven currents and waves, the seawater pH_T differences were damped, while stagnant conditions resulted in a stronger seawater pH_T reduction. The seawater pH_T value for the extreme site ranged from 6.80 to 7.91 with mean values of 7.4 (SD = 0.26).

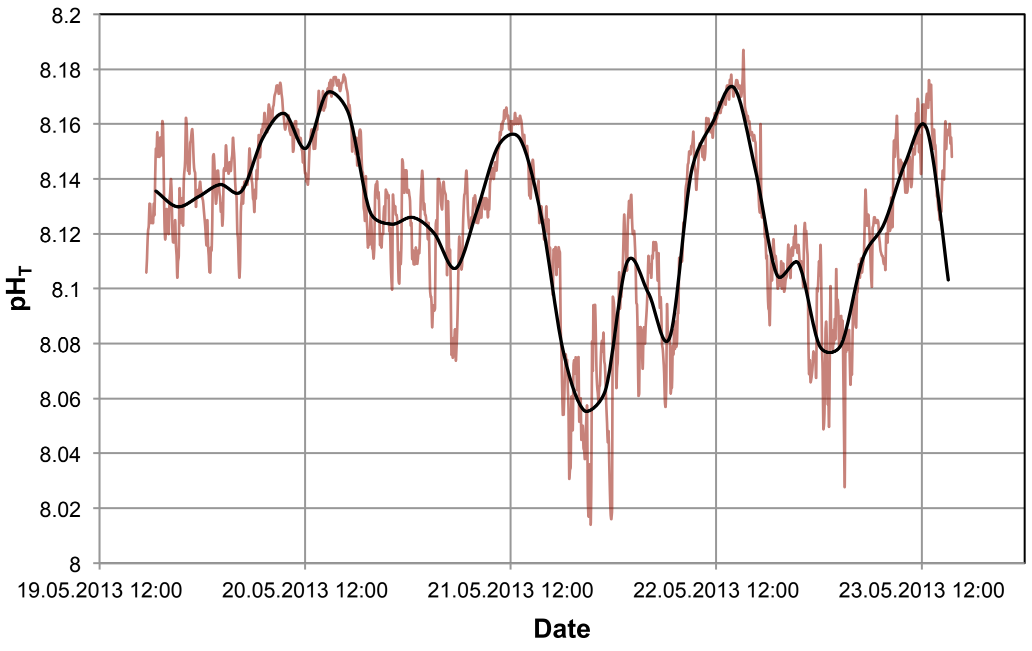

Fig. S3: Seawater pH (pH$_T$) data recorded with a CTD at the control site. The red line shows the individual measurements (one per 5 min), the black line the running mean of 30 data points. pH$_T$ data are displayed in total scale.

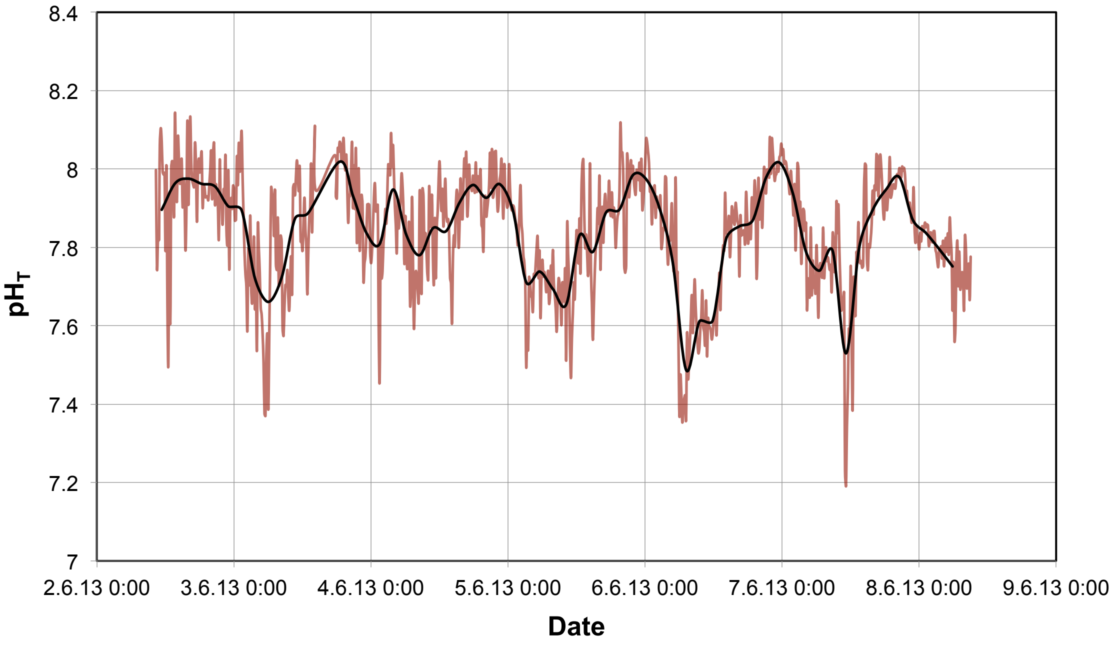

Fig. S4: Seawater pH (pH$_T$) recorded at intermediate site. pH$_T$ values were cross-referenced with CTD deployed at the Control Site. The red line shows the individual measurements (one per 10 min), the black line running mean of 15 data points. pH$_T$ data are displayed in total scale.

Previous monitoring of the Upa Upasina seeps (Farbricius *et al.*[1]) allowed the derivation of a pH map. It shows that the values of their field campaign and the seawater pH$_T$ data obtained for this study (2013, 2014) are in very good agreement. Therefore we are confident that the average values are representative for longer time scales and can be compared with average boron isotopic signatures. The measured total alkalinity (TA, n = 11) collected during the 2013 and 2014 field trip were also in good agreement with

previous measurements[e.g. 1,7], and were used in combination with the measured pH_T to calculate dissolved inorganic carbon (DIC), pCO_2 and the aragonite saturation state (Ω_{arag}). Table S3 summarizes the carbonate chemistry for the four different regions where the coral cores were collected and seawater pH_T was monitored.

Table S3: Seawater carbonate chemistry of the four sites (control site (C), intermediate site (I), low pH site (L) and extreme site (E)) with measured seawater pH (in total scale, pH_T. n = the number of individual pH records and N = the number of logger deployments) and total alkalinity (TA; N = 11). Other carbonate chemistry parameters (dissolved inorganic carbon (DIC), partial pressure of carbon dioxide (pCO_2), saturation state for aragonite (Ω_{arag})) were calculated using the measured variables and the software package seacarb[8]. Values are means, standard deviations in parentheses).

Site	C	I	L	E
**pH_T**	8.1 (0.1)$^{a)}$	7.9 (0.20)$^{b)}$	7.6 (0.17)$^{b)}$	7.4 (0.26)$^{c)}$
**n/N (phT)**	2932 / 3	2148 / 2	3573 / 3	477 / 1
**TA [μmol kg^{-1}]**	2272 (33)	2272 (33)	2272 (33)	2272 (33)
**N (TA)**	11	11	11	11
**DIC_{cal} [μmol kg^{-1}]**	1913	2030	2164	2235
**pCO_{2cal} [μatm]**	335	591	1302	2147
**Ω_{arag}**	4.02	2.82	1.56	1.02

This unique field setting allowed us to verify for the first time the application of $\delta^{11}B$ as a seawater pH_T proxy for tropical corals growing naturally *in situ* under a wide range of seawater pH_T levels. The corals were exposed to the full range of natural environmental heterogeneity in light, currents, temperature and food supply, which distinguishes them from specimens taken from controlled laboratory conditions.

- Average boron isotopic signature, variability and corresponding internal calcifying conditions:

The boron isotopic signature of the coral skeletons differed significantly between the four sites (non-parametric Kruskal Wallis Test, $p<0.001$). Post-hoc pairwise comparisons showed that the corals from the extreme site were significantly different from all other sites. Those from the intermediate site and low pH_T site were not significantly different from each other, nor were the intermediate site and control site, while the low pH_T site was significantly different from the control site (Table S4).

Table S4: P-values derived from post-hoc pairwise comparisons (Chi-squared) of $\delta^{11}B$ and ΔpH between sites (control (C), intermediate site (I), low pH site (L) and extreme site (E)). *** signifies $p < 0.001$, ** $p < 0.01$ and * $p < 0.05$.

$\delta^{11}B$				ΔpH			
**sites**	**C**	**I**	**L**	**sites**	**C**	**I**	**L**
**I**	0.991	-	-	**I**	*	-	-
**L**	**	0.094	-	**L**	***	**	-
**E**	***	***	**	**E**	***	**	0.999

Boron isotopic signatures were converted into internal calcifying pH (pH_{cf}), used to calculate the extent of pH_{cf} up-regulation ($\Delta pH = pH_{cf} - pH_T$). As seawater pH_T declined 0.5 units from the mean control site value of 8.1 to 7.6 at the low pH_T site, the $\delta^{11}B$ decreased by 1.45‰. This suggested a reduction in internal pH_{cf} of only 0.12 (Table S3). The 0.2 unit decrease in seawater pH_T between the low pH_T site and the extreme site

resulted in a 1.7‰ decline of $\delta^{11}B$, suggesting a change in the internal pH_{cf} of 0.18. Hence, this resulted in a relationship between $\delta^{11}B$ and seawater pH_T that deviates from the boron fractionation curve, rather reflecting polynomial response (AIC criteria were used to test for the best fit: linear vs polynomial of 2nd and 3rd rank: 1071 > 1067 < 1068, respectively).

Laboratory studies derived empirical $\delta^{11}B$-pH calibration equations[9,10] for tropical corals that differ between species and suggest different vital effects on $\delta^{11}B$ incorporation. Both studies used different species (in each case two different coral species were tested) to derive such relationship. The shape of the calibrations within a study is similar but off-set between the two species. Comparing the two studies, the calibration relationships deviate from each other (Fig. 1; main manuscript). Likely vital effects on boron incorporation can play a role (which were also shown to differ with skeletal region within the same coral species analyzed[11]). Also different sites of origin of the corals used in the culturing experiment could be essential and contribute to differences in vital effects (in case of Hönisch *et al.*[9] Ruykyus Island, Japan and in case of Krief *et al.*[10] the Red Sea). A recent study showed no effect of seawater pH on $\delta^{11}B$ in massive *Porites* grown at the Heron Island reef flat[12]. This highlights that the response (in particular the shape of the relationship) can be very different. In this latter case it was argued to derive from strong environmental fluctuations on the reef flat at Heron Island and supports the idea that origin can make a difference and determine vital effects.

Table S5: Seawater pH_T and coral skeletal average $\delta^{11}B$ (‰) for the four sites (control site (C), intermediate site (I), low pH_T site (L) and the extreme site (E)) and the individual coral cores (Table S1). Skeletal $\delta^{11}B$ values were converted into internal calcifying fluid pH (pH_{cf}) and pH up-regulation intensity ($\Delta pH = pH_{cf} - pH_T$). Other internal conditions at the site of calcification were adopted from McCulloch et al.[13]: dissolved inorganic carbon at the site of calcification (DIC_{cf}) as 2*DIC of seawater (in μmol kg^{-1} seawater) and calcium concentration [Ca^{2+}] as 0.5 mmol above seawater (ca. 10.5 mmol[1]) resulting in an internal calcium concentration [Ca^{2+}] of 11 mmol. The pH_{cf}, internal DIC_{cf} and [Ca^{2+}] were used to calculate the aragonite saturation state at the site of calcification ($\Omega_{arag\ cf}$). Values are means per colony (standard errors in parentheses). In bold: means and standard errors of multiple colonies.

Site	C	I	L	E
**pH_T**	8.1 (0.001)	7.9 (0.004)	7.6 (0.003)	7.4 (0.012)
**$\delta^{11}B$**	c13) 20.70 (0.40)	I1) 20.00 (0.41)	L1) 20.09 (0.41)	E1) 18.91 (0.31)
	c1A) 20.75 (0.50)	I2) 21.09 (0.39)	L2) 19.62 (0.52)	E2) 17.07 (0.71)
	c2A) 20.64 (0.57)		L3) 18.74 (0.48)	E3) 17.54 (0.51)
	c3A) 21.73 (0.53)			E4) 17.70 (0.53)
	c14) 20.73 (0.43)			
**Mean**	**20.91 (0.26), n = 86, N = 5**	**20.54 (0.54), n = 31, N = 2**	**19.48 (0.40) , n = 54, N = 3**	**17.80 (0.39), n = 75, N = 4**
**pH_{cf}**	c13) 8.22 (0.03)	I1) 8.17 (0.03)	L1) 8.18 (0.03)	E1) 8.08 (0.03)
	c1A) 8.22 (0.04)	I2) 8.25 (0.03)	L2) 8.13 (0.04)	E2) 7.83 (0.08)
	c2A) 8.21 (0.04)		L3) 8.05 (0.05)	E3) 7.92 (0.06)
	c3A) 8.30 (0.04)			E4) 7.94 (0.06)
	c14) 8.22 (0.03)			
**Mean**	**8.24 (0.02)**	**8.21 (0.04)**	**8.12 (0.04)**	**7.94 (0.05)**
**ΔpH**	c13) 0.12 (0.03)	I1) 0.27 (0.03)	L1) 0.58 (0.03)	E1) 0.68 (0.03)
	c1A) 0.12 (0.04)	I2) 0.35 (0.03)	L2) 0.53 (0.04)	E2) 0.43 (0.08)
	c2A) 0.11 (0.04)		L3) 0.45 (0.05)	E3) 0.52 (0.06)
	c3A) 0.20 (0.04)			E4) 0.54 (0.06)
	c14) 0.12 (0.03)			

**Mean**	**0.14 (0.02)**	**0.31 (0.04)**	**0.52 (0.04)**	**0.54 (0.05)**
**DIC_{cf}**	3826	4060	4328	4470
**$[Ca^{2+}]_{cf}$**	11	11	11	11
Ω_{cf}	c13) 11.39 (0.63)<br>c1A) 11.52 (0.79)<br>c2A) 11.39 (0.92)<br>c3A) 12.92 (0.86)<br>c14) 11.47 (0.72)	I1) 11.30 (0.69)<br>I2) 13.17 (0.64)	L1) 12.86 (0.76)<br>L2) 12.11 (0.97)<br>L3) 10.43 (0.87)	E1) 11.41 (0.61)<br>E2) 8.34 (1.34)<br>E3) 8.99 (0.93)<br>E4) 9.29 (1.01)
**Mean**	**11.72 (0.34)**	**12.27 (0.49)**	**11.75 (0.531)**	**9.41 (0.53)**

Based on the assumption that the dissolved inorganic carbon (DIC) concentration is doubled at the site of calcification, the internal aragonite saturation state $\Omega_{arag\ cf}$, the derived mean Ω_{cf} for the extreme site is around 9.41 (s.e.m. 0.53). A recent study argues that corals can concentrate DIC_{cf} within the internal calcifying fluid to foster calcification[14], which is supported by an up-regulation of HCO_3^- transporter genes[15], allowing them to maintain a high Ω_{cf} even at reduced pH_{cf}. This would furthermore enable corals to continue calcifying even at lower pH_{cf} values.

High spatial resolution studies of boron isotopes in corals have revealed pronounced variation in the same genus[16,17] and also other coral species[18]. In this study, $\delta^{11}B$ values were highly variable even at the control site (Fig. S5; average range 7.3 for the control site and of 8.1 for the extreme site) where the seawater pH_T was more stable compared to the other sites (Fig. S3-4). Seep site corals, however, showed few values > 23‰ (corresponding to an internal pH_{cf} of 8.39), in contrast to control site corals with $\delta^{11}B$ of up to 25.7‰ or a pH_{cf} of 8.56.

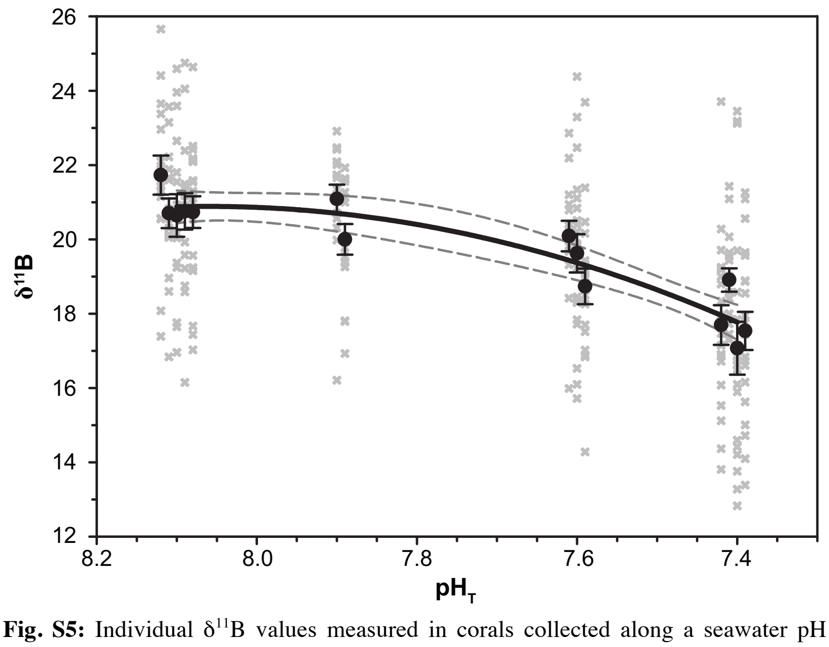

Fig. S5: Individual $\delta^{11}B$ values measured in corals collected along a seawater pH (pH_T total scale) gradient. Black circles and error bars indicate the mean bulk values per colony +/- SE; n=15-20 per site (grey crosses are individual measurements with an internal error: 0.5 1SD). Replicate colonies at various sites (control pH_T = 8.1, intermediate site pH_T = 7.9, low pH site pH_T = 7.6 and extreme site pH_T = 7.4)) are jittered horizontally for clarity.

Such high variations have been previously described for tropical corals, and it was argued that these variations reflect the effect of biological processes on skeletal isotopic composition rather than external seawater pH_T variations[16,17]. Several factors are thought

to contribute to these internal variations in pH_{cf}, but they are not yet fully understood. The general assumption is based on passive diffusion of seawater to the site of calcification and an active elevation of pH_{cf} by Ca-ATPase[e.g. 19–22]. However, at the site of calcification, the DIC might be different from seawater (potentially elevated), and this affects the carbonate saturation state. The type, source and transportation of DIC to the site of calcification is still not fully understood[23]. Elevation of pH_{cf} requires energy. This demand needs to be met, but investment might vary temporally depending on energy availability and energetic trade-offs between other energy-requiring processes. Light is known to enhance the calcification rate[24] and calcification rate is suggested to be linked to internal pH_{cf} up-regulation intensity[13], suggesting that differences in light level can affect the internal pH_{cf}. Hence, days with cloudy conditions might be reflected at the $\delta^{11}B$ level. However, laboratory $\delta^{11}B$ experiments appear to contradict such assumptions, showing no change of $\delta^{11}B$ with differing light levels[25,9], suggesting that the internal pH_{cf} is the same at different light conditions. This raises the question of what other mechanisms could enhance light calcification without affecting internal pH_{cf}? In addition, we also observed boron derived pH_{cf} below seawater values. This can be related to two essential processes: First corals also grow at night. In case of *Porites* from the Papua New Guinea vent sites (subjected to pH of 7.8) these corals still have a not negligible growth at night (almost 40% of day growth[7]) that will contribute to the boron signature. Second the pH surrounding the corals (within their diffusive boundary layer (DBL)) are also lower than seawater pH during the night and internal pH_{cf} values were shown to become even lower within the coral tissue close to the skeleton[21,26–28]. The pH value strongly depends on the flow rate and during rather stagnant conditions pH within the DBL in the night can be really low. Together this can contribute to lower boron isotopic signature in the skeleton. Apart from that we still do not know what factors contribute to the high variability observed in boron isotopic signature. However, this goes beyond the scope of this manuscript. Here we followed the current mainly applied interpretation and application of boron isotopes in coral skeletons.

- Calcification rates:

Relative calcification rates (Table S6) of the individual corals did not change between the control and intermediate sites, based on the corals apparent ability to maintain a similar high internal pH_{cf} (Table S5). Internal pH_{cf} and calcification rate are reduced at the extreme site (Table S6).

Table S6: Calcification rates at the high CO_2 sites, normalized to the mean rate of calcification at the four sites (Control Site (C), Intermediate Site (I), Low pH Site (L) and Extreme Site (E)). Values are means (standard errors in parenthesis)

Site	C	I	L	E
	c13) 0.92 (0.10)	I1) 0.91 (0.10)	L1) 1.18 (0.13)	E1) 0.92 (0.1)
	c1A) 0.97 (0.13)	I2) 1.23 (0.10)	L2) 1.12 (0.18)	E2) 0.73 (0.23)
**Growth ratios**	c2A) 0.97 (0.16)		L3) 0.82 (0.15)	E3) 0.65 (0.12)
	c3A) 1.21 (0.16)			E4) 0.70 (0.16)
	c14) 0.95 (0.11)			
**Mean**	**1.00 (0.05)**	**1.07 (0.16)**	**1.04 (0.11)**	**0.75 (0.06)**

References:

1. Fabricius, K. E. *et al.* Losers and winners in coral reefs acclimatized to elevated carbon dioxide concentrations. *Nat. Clim. Chang.* **1,** 165–169 (2011).
2. Dickson, A. G., Sabine, C. L. & Christian, J. R. *Guide to best practices for ocean CO_2 measurements*. doi:10.1159/000331784 (2007).
3. Fietzke, J. *et al.* Boron isotope ratio determination in carbonates via LA-MC-ICP-MS using soda-lime glass standards as reference material. *J. Anal. At. Spectrom.* **25,** 1953 (2010).
4. Cohen, A. L. & McConnaughey, T. A. Geochemical Perspectives on Coral Mineralization. *Rev. Mineral. Geochemistry* **54,** 151–187 (2003).
5. Nothdurft, L. D. & Webb, G. E. Microstructure of common reef-building coral genera *Acropora*, *Pocillopora*, *Goniastrea* and *Porites*: constraints on spatial resolution in geochemical sampling. *Facies* **53,** 1–26 (2007).
6. Fabricius, K. E., De'ath, G., Noonan, S. & Uthicke, S. Ecological effects of ocean acidification and habitat complexity on reef-associated macroinvertebrate communities. *Proc. R. Soc. B* **281,** 20132479 (2014).
7. Strahl, J. *et al.* Physiological and ecological performance differs in four coral taxa at a volcanic carbon dioxide seep. *Comp. Biochem. Physiol. Part A Mol. Integr. Physiol.* **184,** 179–186 (2015).
8. Lavigne, H. & Gattuso, J. seacarb: seawater carbonate chemistry with R. (2010).
9. Hönisch, B. *et al.* Assessing scleractinian corals as recorders for paleo-pH: Empirical calibration and vital effects. *Geochim. Cosmochim. Acta* **68,** 3675–3685 (2004).
10. Krief, S. *et al.* Physiological and isotopic responses of scleractinian corals to ocean acidification. *Geochim. Cosmochim. Acta* **74,** 4988–5001 (2010).
11. Holcomb, M. *et al.* Coral calcifying fluid pH dictates response to ocean acidification. *Sci. Rep.* **4,** 5207 (2014).
12. Georgiou, L. *et al.* pH homeostasis during coral calcification in a free ocean CO_2 enrichment (FOCE) experiment, Heron Island reef flat, Great Barrier Reef. *Proc. Natl. Acad. Sci. U. S. A.* **112,** 13219–24 (2015).
13. McCulloch, M., Falter, J., Trotter, J. & Montagna, P. Coral resilience to ocean acidification and global warming through pH up-regulation. *Nat. Clim. Chang.* **2,** 623–627 (2012).
14. Allison, N., Cohen, I., Finch, A. a, Erez, J. & Tudhope, A. W. Corals concentrate dissolved inorganic carbon to facilitate calcification. *Nat. Commun.* **5,** 5741 doi:10.1038/ncomms6741 (2014).
15. Vidal-Dupiol, J. *et al.* Genes related to ion-transport and energy production are upregulated in response to CO_2-driven pH decrease in corals: new insights from transcriptome analysis. *PLoS One* **8,** e58652 (2013).
16. Allison, N., Finch, A. A. & EIMF. $\delta^{11}B$, Sr, Mg and B in a modern *Porites* coral: the relationship between calcification site pH and skeletal chemistry. *Geochim. Cosmochim. Acta* **74,** 1790–1800 (2010).
17. Rollion-Bard, C., Chaussidon, M. & France-Lanord, C. Biological control of

internal pH in scleractinian corals : Implications on paleo-pH and paleo-temperature reconstructions. *Comptes rendus - Geosci.* **343,** 397–405 (2011).

18. Blamart, D. *et al.* Correlation of boron isotopic composition with ultrastructure in the deep-sea coral *Lophelia pertusa*: Implications for biomineralization and paleo-pH. *Geochemistry, Geophys. Geosystems* **8,** 1–11 (2007).
19. McConnaughey, T. A. & Whelan, J. F. Calcification generates protons for nutrient and bicarbonate uptake. *Earth-Science Rev.* **42,** 95–117 (1997).
20. Gagnon, A. C., Adkins, J. F. & Erez, J. Seawater transport during coral biomineralization. *Earth Planet. Sci. Lett.* **329-330,** 150–161 (2012).
21. Al-Horani, F., Al-Moghrabi, S. M. & de Beer, D. Microsensor study of photosynthesis and calcification in the scleractinian coral, *Galaxea fascicularis*: active internal carbon cycle. *J. Exp. Mar. Bio. Ecol.* **288,** 1–15 (2003).
22. Furla, P., Galgani, I., Durand, I. & Allemand, D. Sources and mechanisms of inorganic carbon transport for coral calcification and photosynthesis. *J. Exp. Biol.* **203,** 3445–57 (2000).
23. Allemand, D., Tambutté, É., Zoccola, D. & Tambutté, S. Coral calcification, cells to the reef. in *Coral Reefs: An Ecosystem in Transition SE - 9* (eds. Dubinsky, Z. & Stambler, N.) 119–150 (Springer Netherlands, 2011).
24. Comeau, S., Carpenter, R. C. & Edmunds, P. J. Effects of irradiance on the response of the coral Acropora pulchra and the calcifying alga Hydrolithon reinboldii to temperature elevation and ocean acidification. *J. Exp. Mar. Bio. Ecol.* **453,** 28–35 (2014).
25. Dissard, D. *et al.* Light and temperature effects on δ^{11}B and B/Ca ratios of the zooxanthellate coral *Acropora* sp.: results from culturing experiments. *Biogeosciences* **9,** 4589–4605 (2012).
26. Kühl, M., Cohen, Y., Dalsgaard, T., Jergensenl, B. B. & Revsbech, N. P. Microenvironment and photosynthesis of zooxanthellae in scleractinian corals studied with microsensors for O_2, pH and light. *Mar. Ecol. Prog. Ser.* **117,** 159–172 (1995).
27. Chan, N. C. S., Wangpraseurt, D., Kühl, M. & Connolly, S. R. Flow and Coral Morphology Control Coral Surface pH: Implications for the Effects of Ocean Acidification. *Front. Mar. Sci.* **3,** 1–11 (2016).
28. Agostini, S. *et al.* The effects of thermal and high-CO_2 stresses on the metabolism and surrounding microenvironment of the coral *Galaxea fascicularis*. *C. R. Biol.* **336,** 384–91 (2013).
